# Optimization of hepatitis C virus screening strategies by birth cohort in Italy

**DOI:** 10.1111/liv.14408

**Published:** 2020-04-02

**Authors:** Loreta A. Kondili, Ivane Gamkrelidze, Sarah Blach, Andrea Marcellusi, Massimo Galli, Salvatore Petta, Massimo Puoti, Stefano Vella, Homie Razavi, Antonio Craxi, Francesco S. Mennini, PITER collaborating group

**Affiliations:** ^1^ Center for Global Health Istituto Superiore di Sanità Rome Italy; ^2^ Center for Disease Analysis Foundation Lafayette CO US; ^3^ Centre for Economic and International Studies Faculty of Economics University of Rome Tor Vergata Rome Italy; ^4^ Department of Accounting Finance and Informatics Kingston Business School Kingston University London UK; ^5^ Department of Biomedical and Clinical Sciences “L Sacco” University of Milan Milan Italy; ^6^ Gastroenterology and Liver Unit, PROMISE University of Palermo Palermo Italy; ^7^ Department of Infectious Diseases ASST Grande Ospedale Metropolitano Niguarda Milan Italy

**Keywords:** cost‐effectiveness, HCV, screening, WHO targets

## Abstract

**Background and Aims:**

Cost‐effective screening strategies are needed to make hepatitis C virus (HCV) elimination a reality. We determined if birth cohort screening is cost‐effective in Italy.

**Methods:**

A model was developed to quantify screening and healthcare costs associated with HCV. The model‐estimated prevalence of undiagnosed HCV was used to calculate the antibody screens needed annually, with a €25 000 cost‐effectiveness threshold. Outcomes were assessed under the status quo and a scenario that met the World Health Organization's targets for elimination of HCV. The elimination scenario was assessed under five screening strategies.

**Results:**

A graduated birth cohort screening strategy (graduated screening 1: 1968‐1987 birth cohorts, then expanding to 1948‐1967 cohorts) was the least costly. This strategy would gain approximately 144 000 quality‐adjusted life years (QALYs) by 2031 and result in an 89.3% reduction in HCV cases, compared to an 89.6%, 89.0%, 89.7% and 88.7% reduction for inversed graduated screening, 1948‐77 birth cohort, 1958‐77 birth cohort and universal screening, respectively. Graduated screening 1 yielded the lowest incremental cost‐effectiveness ratio (ICER) of €3552 per QALY gained.

**Conclusions:**

In Italy, a graduated screening scenario is the most cost‐effective strategy. Other countries could consider a similar birth cohort approach when developing HCV screening strategies.

## Abstract 2

See Editorial on Page 1538

**Abbreviations**
AIFA: Agenzia Italiana del Farmaco (The Italian Medicines Agency)
Comp. cirrhosis: compensated cirrhosis
DAA: direct‐acting antiviral
DCC: decompensated cirrhosis
EUR: euros
GHSS: Global Health Sector Strategy
HCC: hepatocellular carcinoma
HCV: hepatitis C virus
ICER: incremental cost‐effectiveness ratio
LRD: liver‐related death
LTx, subs years: liver transplant, subsequent years
LTx: liver transplant
PCR: polymerase chain reaction
PWID: persons who inject drugs
QALY: quality‐adjusted life year
RNA: ribonucleic acid
SVR: sustained virologic response
UI: uncertainty interval
US: United States
WHO: World Health Organization
WTP: willingness to pay

**Key points**

In 2016, the World Health Assembly passed a resolution to eliminate hepatitis infection by 2030 and the World Health Organization (WHO) introduced global targets for the care and management of HCV infection, known as the Global Health Sector Strategy (GHSS) goals for hepatitis. The lack of available patients linked to care and available to treat, however, remains the key bottleneck for several countries aiming to achieve HCV elimination. The data reported in this study are of importance in that they demonstrate the cost‐effective profiles of several HCV screening strategies in the general population of a high‐endemic country. Differentiated screening strategies based on epidemiological peculiarities of HCV infection can support increases in diagnosis and subsequent treatment of infected patients necessary for realizing elimination.

## 1 INTRODUCTION

Hepatitis C virus (HCV) is a leading cause of liver‐related morbidity and mortality, causing an estimated 71 million infections globally.^1^ The size of the infected population and the risk of severe complications make HCV a serious public health problem.^2^ However, the use of direct‐acting antiviral (DAA) therapy regardless of fibrosis stage is the current standard of care in many high‐income countries. Thus, the limitation of HCV therapy is no longer treatment efficacy or adherence, but the identification of available patients to treat. Achieving the World Health Organization (WHO)'s Global Health Sector Strategy (GHSS) goals for the elimination of HCV by 2030^3^ has reinvigorated public health initiatives aimed at identifying patients with the disease. Studies originally suggested finding and diagnosing populations at risk for the acquisition and transmission of HCV. While targeted screening program for high‐risk populations, such as injection drug users, are necessary for the elimination of HCV,^4^, ^5^ more is needed to identify what increases in diagnosis are necessary in the general population of high‐endemic countries for achieving elimination.

Italy has been considered the European country with one of the largest burdens of HCV in the general population, with the highest prevalence in the older population and decreasing risk in younger populations.^6^, ^7^ The HCV prevalence in the country is approximately 1%, though previous studies have estimated rates as high as 7% in those born between 1935 and 1944, while those aged 30 years and younger are at less risk of acquiring HCV.^7^ A large number of infections occurred from the 1950s to the 1960s via iatrogenic transmission due to the use of unsterilized materials.^6^, ^7^ More so, there are geographical differences in prevalence distribution. The highest rates of HCV have been reported in Southern Italy, where the HCV prevalence in younger cohorts is quite limited.^6^, ^7^, ^8^ Considering the natural history of chronic HCV infection and the wide use of antiviral therapy in Italy, total HCV cases still remain higher than in other European countries, such as Spain and France.^9^ With more than 56 000 patients treated in 2018, Italy has taken substantial strides in managing its HCV disease burden. However, the number of HCV‐infected individuals available to treat is estimated to run out by 2025 given the current treatment rates, leaving a large proportion of individuals with the potential to progress to later stage liver disease.^8^ Thus, cost‐effective screening strategies are needed to make elimination a reality in Italy. We aimed to determine the cost‐effectiveness of expanded HCV screening strategies among different population cohorts in Italy.

## 2 MATERIALS AND METHODS

### 2.1 Study design

An Excel‐based Markov disease burden model^1^ was populated with Italian data to quantify the annual HCV‐infected population by liver disease stage, sex and age.^8^ The model simulates the natural history of the disease and forecasts disease burden, medical costs and health effects of HCV, assessed under the status quo and a scenario to achieve the WHO's GHSS targets (80% reduction in incidence of chronic HCV infections between 2015 and 2030, 65% reduction in HCV‐related deaths due to chronic HCV infection between 2015 and 2030, 90% diagnosis coverage of the HCV‐infected population in 2015 and 80% treatment coverage of the eligible HCV‐infected population in 2015)^3^ considering five screening strategies:

1. Status quo: only at‐risk populations screened in Italy,^2^
2. Targeted general population screening for the 1948‐77 birth cohort,^8^ ^1^These two screening strategies are based on a previous modelling study,^8^ which estimated more than 70% of infected (F0‐F3) individuals were within the 1948‐1978 birth cohorts by 2020.
3. Targeted general population screening for the 1958‐77 birth cohort,^8^
4. Graduated birth cohort screening 1 (birth cohorts 1968‐1987 beginning in 2020 to identify young populations at risk for transmitting HCV, expanding to the 1948‐1967 birth cohort beginning in 2023 to identify older populations before their disease advances),
5. Graduated birth cohort screening 2 (birth cohorts 1948‐1967 beginning in 2020 to identify older populations before their disease advances, and the younger birth cohort 1968‐1987 at risk for transmitting HCV beginning in 2023),
6. Universal screening: The entire Italian population was considered to be screened.

### 2.2 Input parameters

The Italian HCV‐infected population, with or without a prior HCV diagnosis,^7^ disease burden,^8^ cost (in euros)^10^, ^11^ and health‐related quality of life measures^12^ was obtained from the recently published literature (Table 1). The number of patients treated with DAAs from 2015 to 2018 was available through the Agenzia Italiana del Farmaco (AIFA, The Italian Medicines Agency).^13^ The per‐patient cost associated with implementing each screening strategy was calculated, considering the proportion of persons who inject drugs (PWID) in each cohort. Higher implementation costs were assumed for identifying cases among PWID (€55 per person screened) relative to the general population (€15 per person screened). The estimated proportion of PWID in each cohort is calculated and summarized in Appendix S2. Briefly, a standardized mortality ratio was applied to the 15‐ to 44‐year‐old HCV‐infected population, considering the proportion of the HCV‐infected population that is actively injecting. Using this estimated proportion, the average screening cost per person, per scenario, was calculated (Appendix S2). Prevalence of asymptomatic HCV infections not yet linked to care was used to calculate the number of HCV antibody screens needed annually to diagnose one case, as described in Appendix S1.

**TABLE 1:** Direct medical cost and health effect input parameters

	Parameter	Base	Distribution	First parameter (*α* for beta, shape for gamma, minimum for beta‐PERT)	Second parameter (*β* for beta, scale for gamma, maximum for beta‐PERT)	Reference
Diagnostic and treatment costs (€)	Total screening cost, low‐risk groups^a^	15.00	Beta‐PERT	7.50	30.00	Sum of price of HCV antibody test^b^ and per‐screen implementation cost^c^, assuming half the total cost as minimum and double the total cost as maximum
	Total screening cost, high‐risk groups^a^	55.00	Beta‐PERT	27.50	110.00	Sum of price of HCV antibody test^b^ and per‐screen implementation cost^c^, assuming half the total cost as minimum and double the total cost as maximum
	RNA test/PCR	63.01	—	—	—	Ministero della Salute 2013 (code 91.19.3)
	Genotyping	77.47	—	—	—	Ministero della Salute 2013 (code 91.20.2)
	Fibroscan	50.00	—	—	—	10, 11
	Lab costs	50.00	—	—	—	10, 11
	Antiviral treatment	4000.00	—	—	—	10, 11
Healthcare costs (€)	Fibrotic (F0‐F3)	—	Gamma	100.00	2.77	10, 11
	Comp. cirrhosis	—	Gamma	100.00	8.76	10, 11
	DCC	—	Gamma	100.00	66.26	10, 11
	HCC	—	Gamma	100.00	128.96	10, 11
	LTx, first year	—	Gamma	100.00	737.74	10, 11
	LTx, subs years	—	Gamma	100.00	23.65	^10^
	Death	—	—	—	—	^11^
	Post‐SVR monitoring for cirrhotic patients	50.00	—	—	—	Cost of one ultrasound
QALY utilities, pre‐SVR	Fibrotic (F0‐F3)	—	Beta	11.12	1.52	^12^
	Comp. cirrhosis	—	Beta	16.17	3.31	^12^
	DCC	—	Beta	26.27	9.72	^12^
	HCC	—	Beta	46.47	41.21	^12^
	LTx, first year	—	Beta	26.27	9.72	^12^
	LTx, subs years	—	Beta	26.27	9.72	^12^
	Healthy	1	—	—	—	^12^
QALY utilities, post‐SVR	Fibrotic (F0‐F3)	1	—	—	—	^12^
	Comp. cirrhosis	Same as pre‐SVR				
	DCC	Same as pre‐SVR				
	HCC	Same as pre‐SVR				
	LTx, first year	Same as pre‐SVR				
	LTx, subs years	Same as pre‐SVR				

Abbreviations: comp. cirrhosis, compensated cirrhosis; DCC, decompensated cirrhosis; HCC, hepatocellular carcinoma; HCV, hepatitis C virus; LTx, subs years, liver transplant, subsequent years; LTx, liver transplant; PCR, polymerase chain reaction; QALY, quality‐adjusted life year; RNA, ribonucleic acid; SVR, sustained virologic response.

^a^ Uncertainty in input considered in the most cost‐effective scenario.

^b^ Assumption and [^18^].

^c^ Assumption.

### 2.3 Sensitivity analysis

Deterministic and probabilistic sensitivity analyses were conducted for each scenario to identify the drivers in the model that accounted for the greatest variation in the incremental cost‐effectiveness ratio (ICER) and to generate 95% uncertainty intervals (UIs) around the ICER, given uncertainties in model parameters, using Crystal Ball, a Microsoft Excel (Microsoft Corporation) add‐in by Oracle (Oracle Corporation). In accordance with the International Society of Pharmacoeconomics and Outcomes Research,^14^ costs were assumed to be gamma‐distributed and quality‐adjusted life year (QALY) utilities were assumed to be beta‐distributed (Table 1). Uncertainty in starting prevalence was not considered, since the ICER was calculated relative to the status quo, and the starting prevalence under the status quo would be the same as the prevalence under a screening scenario. Finally, to determine the impact of uncertainty in the cost of screening on the variation in ICER, deterministic and probabilistic sensitivity analyses were conducted for the scenario found to be the most cost‐effective, assuming a beta‐PERT‐distributed cost of screening among both low‐ and high‐risk groups, with a minimum of half the base‐case price and a maximum of double the base‐case price (Table 1).

### 2.4 Role of the funding source

This study was supported by the Italian Ministry of Health, grant number RF‐2016‐02364053 and by a Research Grant from the University of Tor Vergata Rome. The funding source had no role in the study design, the collection, analysis and interpretation of the data, in the writing of the report and in the decision to submit the paper for publication.

## 3 RESULTS

**FIGURE 1:**
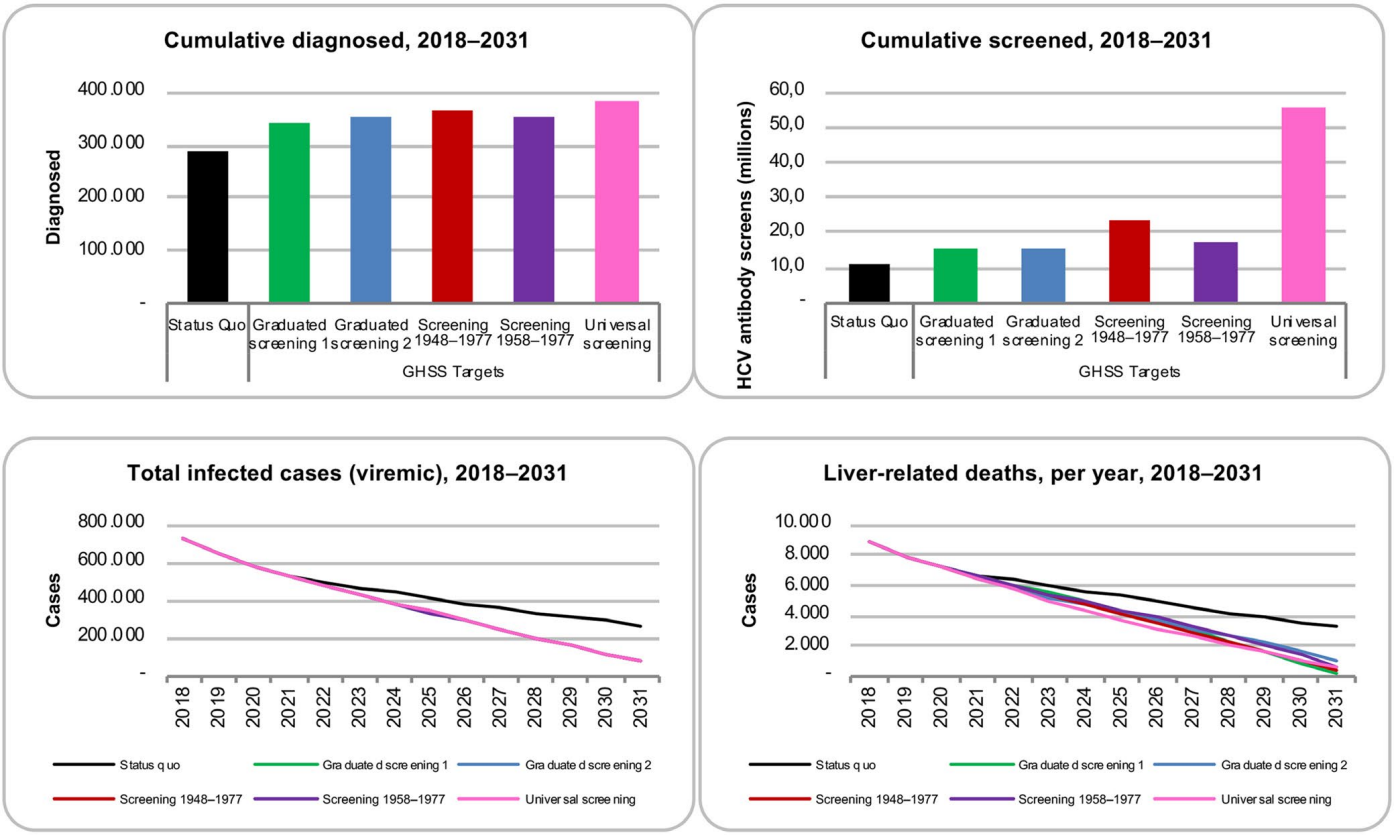
Cumulative diagnosed patients and screens, as well as modelled viraemic cases and liver‐related deaths, by scenario, 2018‐2031. GHSS, Global Health Sector Strategy; HCV, hepatitis C virus

Under the status quo, 290 400 persons would be diagnosed and linked to care, corresponding to 11.3 million screening tests (Figure 1; Table 2). Additionally, 309 200 patients would be initiated on treatment between 2018 and 2031. Total viraemic infections and liver‐related deaths (LRDs) would decline 65% by 2031 (Figure 1). Although this meets the WHO target for a reduction in LRDs, Italy would not achieve the incidence and diagnosis targets.

**TABLE 2:** Scenario inputs, 2017‐2031

Status quo	2017	2018	2019	2020	2021	2022	2025+
Treated	45 000	56 400	44 600	33 700	23 200	15 100	15 100
Newly diagnosed/newly linked to care	30 400	30 400	20 ,000	20 000	20 000	20 000	20 000
Fibrosis score restriction	≥F0	≥F0	≥F0	≥F0	≥F0	≥F0	≥F0
New infections	8200	7500	6700	6100	5600	5200	5200
Treated ages	15+	15+	15+	15+	15+	15+	15+
SVR	95%	95%	98%	98%	98%	98%	98%

GHSS elimination	2017	2018	2019	2020	2021	2022	2025+
Treated	45 000	56 400	44 600	35 700	35 700	36 700	38 000
Incremental newly diagnosed/newly linked to care	—	—	10 400	13 400	13 400	15 400	16 400
Fibrosis score restriction	≥F0	≥F0	≥F0	≥F0	≥F0	≥F0	≥F0
New infections	8200	7500	6700	5500	5000	4100	2600
Treated ages	15+	15+	15+	15+	15+	15+	15+
SVR	95%	95%	98%	98%	98%	98%	98%

Abbreviations: GHSS, Global Health Sector Strategy; SVR, sustained virologic response.

A WHO target scenario (Table 2) was assessed under five different screening strategies. Under these scenarios, between 15.0 million and 55.4 million screening tests would be performed to diagnose between 340 400 and 385 300 persons (Figure 1). Under all screening scenarios, 548 500 persons would start treatment between 2018 and 2031, resulting in an accelerated drop in the number of viraemic cases and LRDs (11 100‐15 300 LRDs averted) by 2031, relative to the status quo. The screening scenarios result in an 89.3%, 89.6%, 89.0%, 89.7% and 88.7% reduction in HCV cases for graduated screening 1, graduated screening 2, 1948‐77 birth cohort, 1958‐77 birth cohort and universal screening, respectively.

**FIGURE 2:**
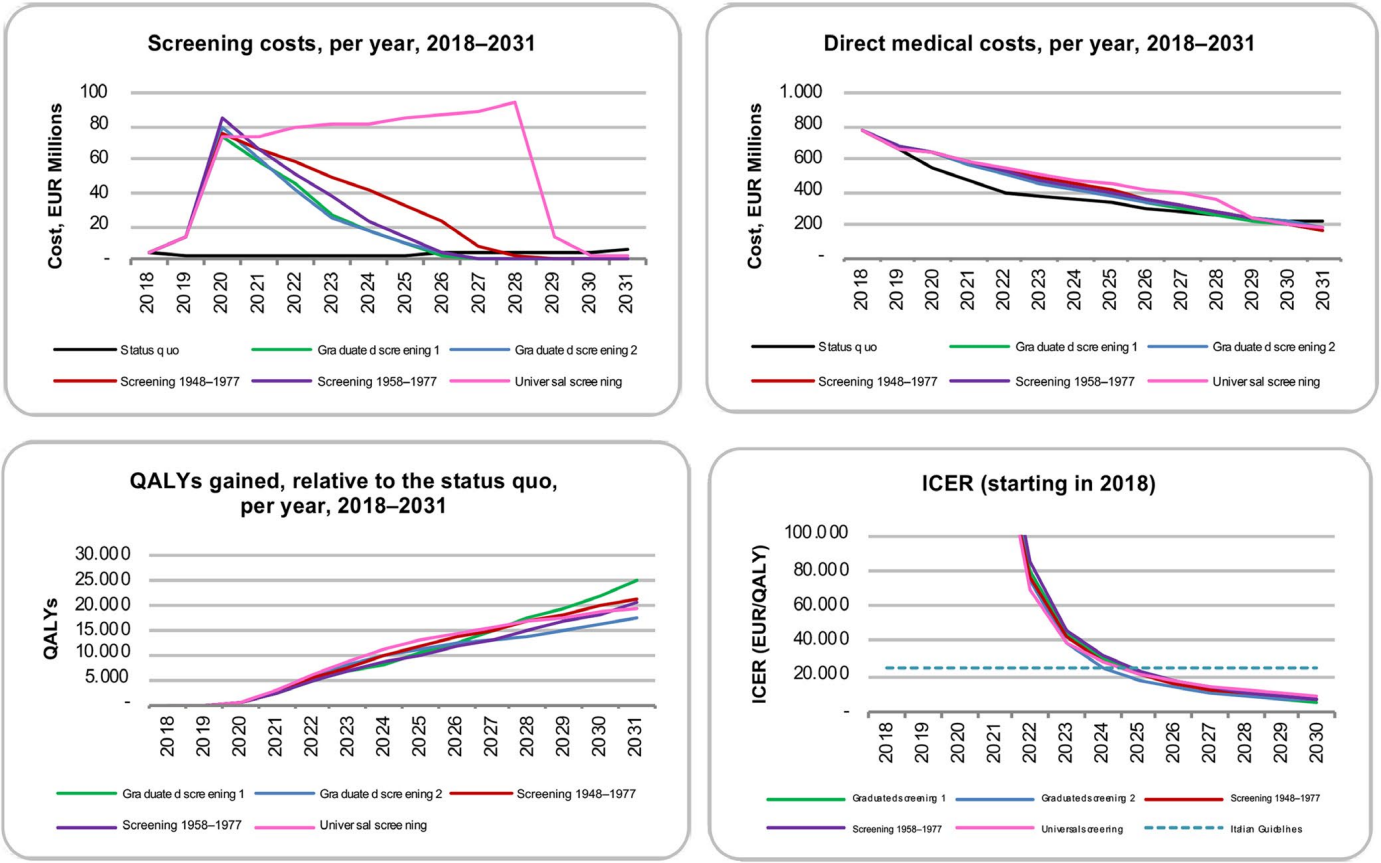
Economic impact of scenarios as measured by screening costs, direct medical costs, QALYs gained relative to the status quo and 13‐year ICER, by scenario, 2018‐2031. EUR, euros; ICER, incremental cost‐effectiveness ratio; QALYs, quality‐adjusted life year

The results of the cost‐effectiveness analysis are presented in Table 3 and shown in Figure 2. Under the status quo, annual screening costs would increase 55%, from €3.4 million in 2018 to €5.3 million by 2031. Annual direct medical costs would show a 70% reduction, from €783.5 million in 2018 to €214.1 million by 2031, cumulating to €5.5 billion over the study period.

**TABLE 3:** Direct medical costs and health effects, by scenario, 2018‐2031

Scenario		Cost (€ millions), 2018‐2031	QALYs gained, 2018‐2031	ICER relative to status quo (€/QALY)			ICER relative to previous least costly scenario (€/QALY)
Status quo		5463	–	–			
GHSS Targets	Graduated screening 1	5974	144 000		3552	3552	
	Graduated screening 2	6028	125 000		4532	^a^	
	Screening 1948‐1977	6081	142 000		4349	^a^	
	Screening 1958‐1977	6083	128 000		4831	^a^	
	Universal screening	6441	145 000		6758	562 855	

Note

Values have been rounded, so ICERs may not be reproducible using table values.

Abbreviations: ICER, incremental cost‐effectiveness ratio; QALY, quality‐adjusted life year; GHSS: Global Health Sector Strategy.

^a^ Strongly dominated scenario (costlier and less effective than another scenario).

Compared to the status quo, all screening scenarios were found to be highly cost‐effective, with an ICER of €3552/QALY (95% UI 1486‐6114) for graduated screening 1, €4349/QALY (95% UI 2265‐7014) for screening the 1948‐77 birth cohort, €4532/QALY (95% UI 2102‐7610) for graduated screening 2, €4831/QALY (95% UI 2470‐7973) for screening the 1958‐1977 birth cohort and €6758/QALY (95% UI 4589‐9481) for universal screening. The graduated screening 1 scenario was the least costly, with €6.0 billion in total direct medical costs by 2031. This was €54.3 million less than graduated screening 2, €107.4 million less than screening in the 1948‐77 birth cohort, €109.1 million less than screening in the 1958‐1977 birth cohort and €467.1 million less than universal screening. Relative to the status quo, graduated screening 1 would gain approximately 144 000 QALYs by 2031, compared to 125 000, 145 000, 142 000 and 128 000 QALYs for graduated screening 2, universal, 1948‐1977 birth cohort and 1958‐77 birth cohort screening, respectively. Excluding the three scenarios that were costlier and less effective than graduated screening 1, universal screening yielded an ICER of €562 855 per QALY relative to graduated screening 1.

**FIGURE 3:**
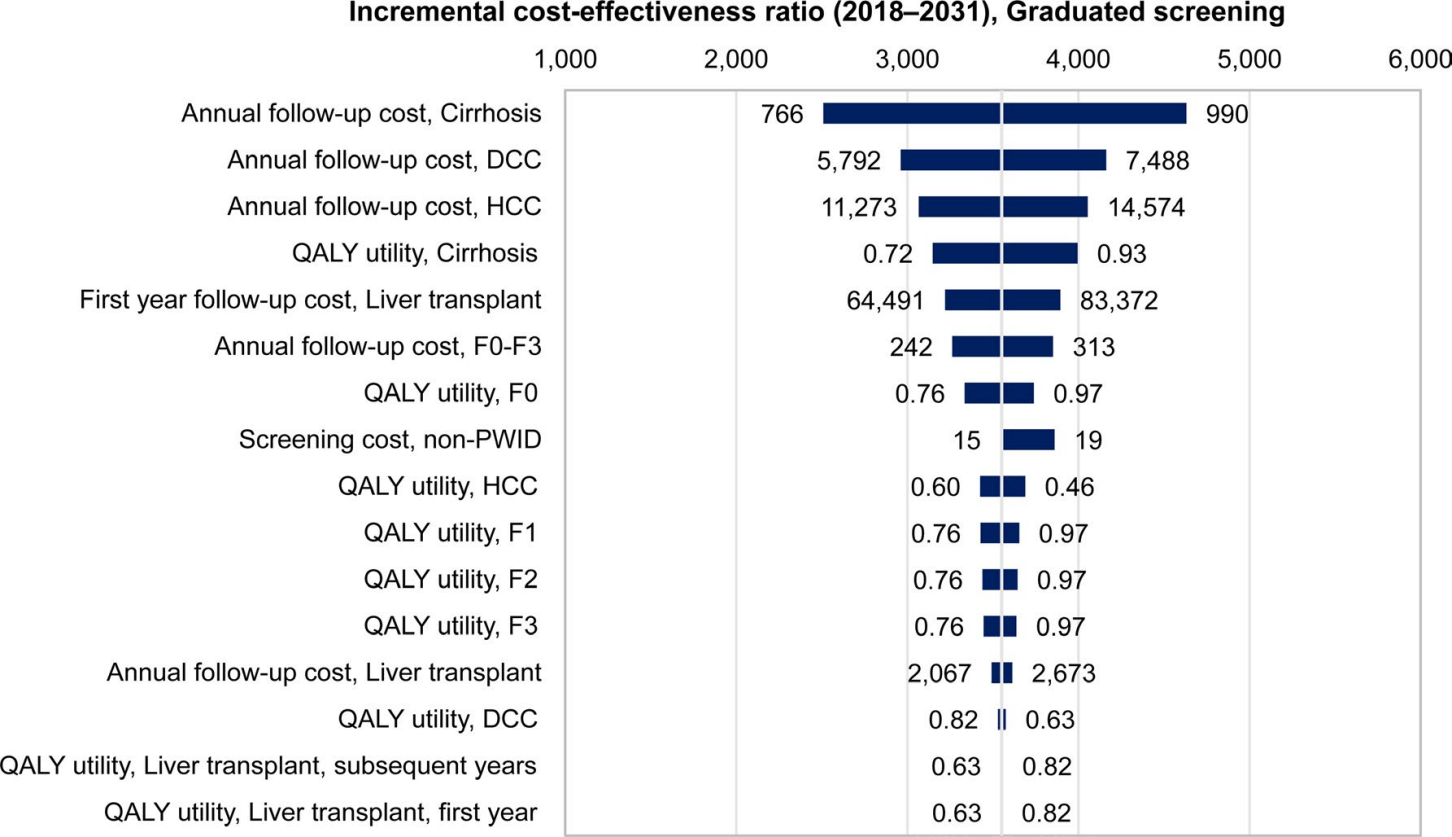
One‐way sensitivity analysis for the 2018‐2031 incremental cost‐effectiveness ratio, graduated screening 1 scenario. The central vertical line in the tornado diagram represents all parameters at base values and the base 2018‐2031 cost‐effectiveness ratio for the graduated screening 1 scenario. The horizontal bars represent the variation in the incremental cost‐effectiveness ratio given the variations in the parameters of the scenario. The variables are arranged by explained variation in the incremental cost‐effectiveness ratio, with ‘Annual follow‐up cost, Cirrhosis’ being the most impactful. DCC, decompensated cirrhosis; HCC, hepatocellular carcinoma; QALY, quality‐adjusted life year

**FIGURE 4:**
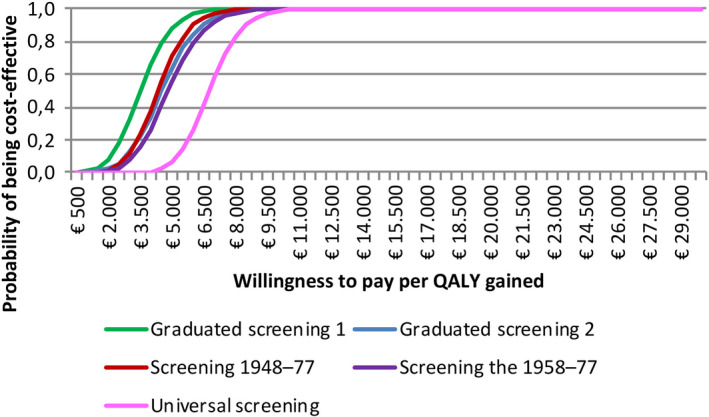
Cost‐effectiveness acceptability curve, by scenario. The cost‐effectiveness acceptability curve shows the proportion of Monte Carlo simulations resulting from varying cost and QALY (quality‐adjusted life year) parameters that are cost‐effective given a willingness to pay threshold. QALY, quality‐adjusted life year

One‐way sensitivity analysis showed that more than 90% of the variation in the 2018‐2031 ICER was due to the annual follow‐up costs of cirrhosis, decompensated cirrhosis, hepatocellular carcinoma, the QALY utility for cirrhosis and the cost of liver transplantation (Figure 3). Since the choice of scenario does not make a difference in the ranking of uncertain parameters by explained variation in the ICER, only results for the graduated screening scenario 1 were reported. The probabilistic sensitivity analysis revealed that, at a willingness to pay (WTP) threshold of €25 000 per QALY gained, all scenarios were cost‐effective 100% of the time (Figure 4).

**FIGURE 5:**
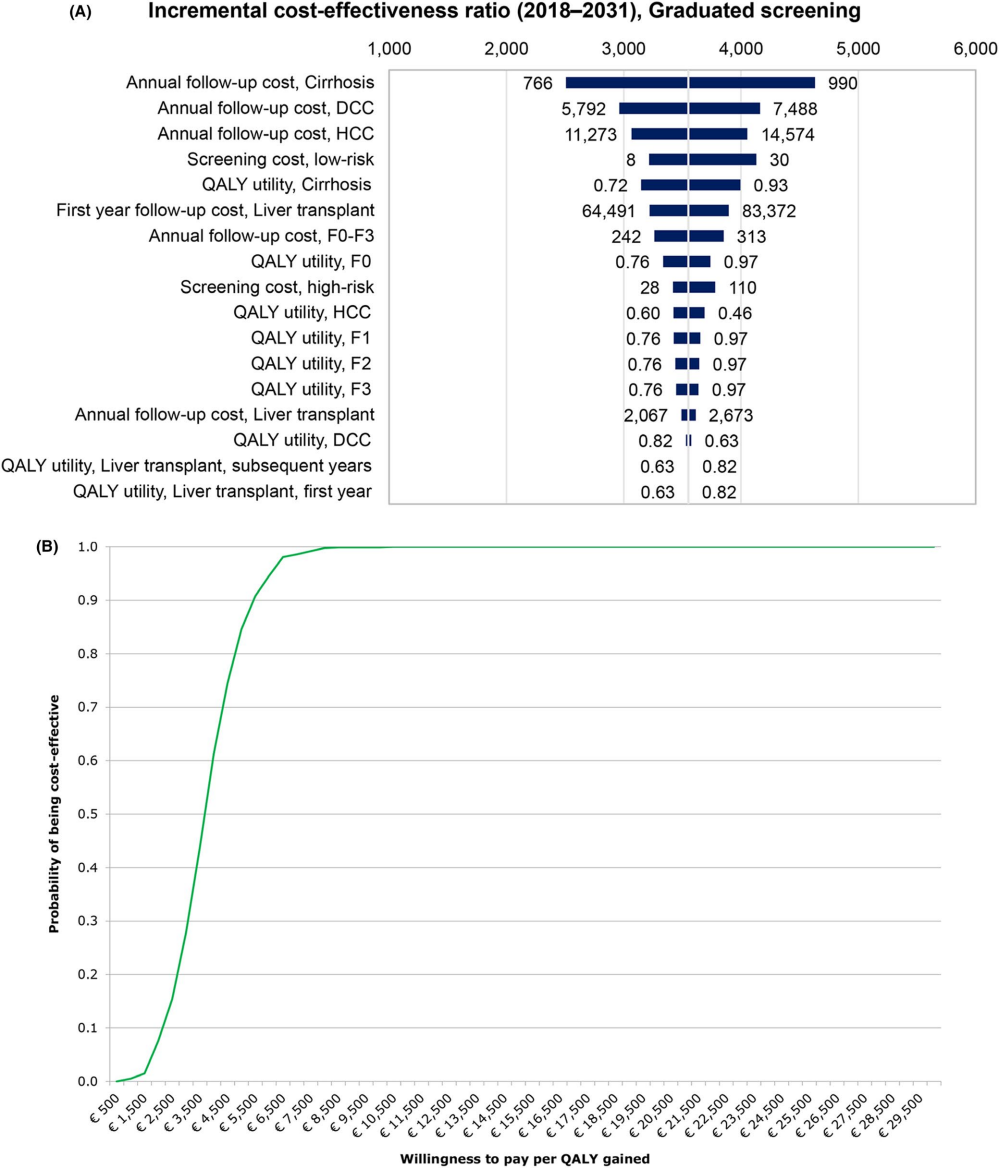
A, One‐way sensitivity analysis for the 2018‐2031 incremental cost‐effectiveness ratio, including uncertainty in cost of screening, graduated screening 1 scenario. B, Cost‐effectiveness acceptability curve, including uncertainty in cost of screening, graduated screening 1 scenario*.* DCC, decompensated cirrhosis; HCC, hepatocellular carcinoma; QALY, quality‐adjusted life year

Allowing for uncertainty (ie varying the price from half to up to double the assumed base price of screening) in the cost of screening among both low‐ and high‐risk groups, graduated screening 1, which was the most cost‐effective scenario relative to the status quo, had an ICER of €3552/QALY (95% UI 1570‐6359). One‐way sensitivity analysis revealed that more than 90% of variation in the ICER was mainly due to the annual follow‐up costs of cirrhosis, decompensated cirrhosis, hepatocellular carcinoma, followed by the cost of screening among low‐risk groups, the QALY utility for cirrhosis and the cost of liver transplantation (Figure 5A). The probabilistic sensitivity analysis showed that at a WTP of €25 000, graduated screening 1 remained cost‐effective 100% of the time (Figure 5B).

## 4 DISCUSSION

This study aimed to evaluate the cost‐effectiveness of five potential HCV screening strategies in Italy. The universal screening and birth cohort screening scenarios, which achieve all targets of the HCV elimination goals, were found to be cost‐effective when compared to the status quo scenario, suggesting a coordinated screening program may be beneficial in moving Italy towards elimination of HCV.

### 4.1 Birth cohort screening

We evaluated the cost‐effectiveness of screening in the birth cohorts 1948‐1988, as they were previously identified as having the highest prevalence of undiagnosed HCV infection.^8^ Screening the 1948‐77 birth cohort would result in fewer QALYs gained compared to the universal and graduated screening 1 scenario and cost less than the universal screening scenario, but more than the graduated screening strategies. Other studies also found that targeted screening among sub‐populations with high HCV prevalence is cost‐effective.^15^, ^16^ In a previous meta‐analysis of all available cost‐effectiveness studies before the availability of DAAs, the cost per QALY gained of screening programs among asymptomatic cohorts at general risk for HCV ranged between US $4200 and $50 000. This was much lower than focusing on specific risk groups, which had an estimated ICER of between $848 and $128 424 per QALY gained. In the study, age of the target population to be screened and prevalence were the main drivers of cost‐effectiveness.^17^ These are also important drivers for developing screening strategies in Italy.^18^ Despite the lower rates of drug use across Italy compared to other countries, this risk group represents the most recent wave of new infections,^19^ which in addition to other high risk populations as imnates and migrants are mainly distributed among the 1968‐1988 birth cohorts,^20^, ^21^, ^22^ who are asymptomatic. Without including these groups in specific screening policies, continued disease burden is expected. Additionally, results of the sensitivity analysis, which examined the cost‐effectiveness of each strategy using a €25 000/QALY WTP threshold, remained high (*P* > 99%) once the uncertainty in parameters was considered. Parameters relating to the disease state costs and health outcome utilities were found to have the highest impact on the cost‐effectiveness results.

### 4.2 Graduated screening

Consequently, the graduated screening strategies aim to capture individuals who may be at a higher risk for HCV but are currently asymptomatic. The graduated screening 1 scenario, which identifies young populations at risk for transmitting HCV before expanding to identify older populations before their disease advances, was the least costly screening strategy, with €6.0 billion in direct medical costs by 2031. Relative to the status quo, graduated screening 1 would gain approximately 144 000 QALYs by 2031, which was more than the other birth cohort strategies and also produced a lower ICER. The graduated screening 2 scenario, which first identifies older populations before their disease advances and then screens younger cohorts at risk for transmitting HCV, was also less costly (€6.0 billion), but had fewer QALYs gained (125 000) compared to graduated screening 1 scenario. From a disease burden perspective, both graduated screening strategies have significant impact on overall reduction in total viraemic infections and liver‐related mortality by 2031 (Figure 1), though graduated screening scenario 1 results in more QALYs gained because it is more likely to identify asymptomatic individuals early and prevent the progression of liver disease. Additionally, both graduated scenarios were found to be highly cost‐effective (as seen in Table 3). Even upon allowing for a wide variation in the cost of screening among both low‐ and high‐risk groups, the graduated screening 1 scenario remained highly likely (*P* > 99%) to be cost‐effective at the €25 000/QALY WTP threshold (Figure 5B). Screening in this younger cohort would likely detect individuals at higher risk of infectiousness, decreasing the potential to transmit new infections compared to screening older patients who are more likely already identified and less likely to contribute to further disease burden.

### 4.3 Programmatic considerations

It is important for policymakers to consider not only the cost‐effectiveness of such strategies, but also their implementation and sustainability. Though the universal screening strategy is recently recommended,^23^, ^24^ it requires higher initial up‐front investment, while the graduated strategy 1 would result in a similar number of QALYs gained and have the largest decline in LRDs and associated mortality, limiting the initial cost of the investment. Other high‐income countries have reported the opposite, where the epidemiology of chronic HCV infection differs from the situation in Italy.^6^, ^7^, ^23^ In the United States, for example, a larger proportion of new HCV transmission is driven by injection drug use among younger populations, meaning universal screening would capture these individuals who are likely asymptomatic.^25^ In Italy, risk‐based HCV testing should be implemented independently by birth cohort. Those aged 30 years and younger without high‐risk behaviours, however, are at less risk of acquiring HCV infection.^7^ Therefore, a graduated screening strategy in the general population, which captures both groups, is recommended, as it is immediately cost‐effective without high initial costs like the universal screening strategy.

### 4.4 Limitations

This study has several limitations that could affect the robustness of the model and the impact of the results. This model does not dynamically estimate new infections and reinfections, nor does it focus on treatment as prevention. However, the current treatment rate in Italy and the need to expand such treatment levels in order to achieve the GHSS targets exceed the proportion required for treatment when compared to other transmission‐based models.^26^ Next, the cost‐effectiveness of a screening strategy is strongly linked to the patient's disease stage at the time of treatment initiation. If treatment is delayed and only patients with severe fibrosis are screened, those with more severe liver disease will accrue higher costs, reducing the impact on QALYs. Additionally, the true cost of DAA treatment in Italy is unknown. A previous analysis estimated that at €4000, treating all patients compared to treating symptomatic individuals would be both cost‐effective and cost‐saving.^12^ Subsequently, this was assumed a reasonable estimate for the cost of therapy. As discussed in a recent systematic review, analyses should consider this rapidly varying cost of DAAs;^27^ however, drug price was not found to be an important factor in the sensitivity analysis. Further, this study partially considered the related costs of healthcare management and reliability of screening tests. In particular, the adequate organizational, health system‐level and operational costs regarding screening of HCV are only partially considered in this study. While diagnosed and immediate initiation of DAA therapy would be the most efficient strategy, screening strategies which further consider the cost for general practitioners to screen and diagnosis HCV patients, though included empirically here, have not yet been fully evaluated. Thus, this modelling work requires real‐life validation in different Italian regions. One such validation study has been carried out in another high‐income, European country. Recent findings from a population‐based, cross‐sectional study in Spain found that a stepwise screening strategy, similar to the graduated screening 1 scenario described here, was cost saving,^28^ supporting that this type of strategy may be feasible. Though graduated or birth cohort screening is less costly than universal screening, further examinations of each scenario's associated programmatic costs should be evaluated in terms of their impact on the Italian Health Sanitary budget and their sustainability.

## 5 CONCLUSIONS

Holistic screening strategies for hepatitis C should be implemented, considering the prevalence, the reliability of diagnostic assays, the natural history of infection, the benefits and risks of therapeutic intervention and the potential benefits to society. Universal screening and birth cohort screening scenarios, which achieve all targets of the HCV elimination goals, were found to be cost‐effective when compared to the status quo scenario in Italy, suggesting a coordinated screening program may be beneficial in moving Italy towards elimination of HCV. A graduated screening strategy has both clinical and economic benefits to the population and could sustain Italy's momentum towards achieving the HCV elimination goals. Other countries, particularly those which may not have the economic or structural means to implement universal screening but are interested in developing screening strategies based on specific HCV epidemiology, could consider a birth cohort approach based on specific epidemiological data and real‐life treatment rates.

## CONFLICT OF INTEREST

The authors declare no conflict of interest.

## Supporting information

Appendix S1‐S2
