# Supplementary material for: Optimization of hepatitis C virus screening strategies by birth cohort in Italy
Source: Liver Int. 2020 Apr 2;40(7):1545–55. doi: 10.1111/liv.14408 (PMC7384106; doi:10.1111/liv.14408)
Supplement: Supplementary file 1 — Appendix S1‐S2 [file LIV-40-1545-s001.docx]

**Appendix 1**

**Description of the screening module for HCV HPT**

To calculate the number of screens necessary for diagnosing a given number of HCV-infected cases, a screening module was developed. The screening module used the inputs and assumptions of the model (annual population by age, number of annual new diagnoses) in conjunction with the outputs (undiagnosed HCV-infected population that was either asymptomatic or not linked-to-care) to calculate the number of annual HCV antibody and HCV RNA tests necessary for a given scenario. Only cases that were either asymptomatic or not yet linked to care were considered as part of the case-finding algorithm. It was assumed that cases with advanced liver disease were symptomatic and would seek care with or without a screening strategy. Therefore, each newly diagnosed case of advanced liver disease would require one HCV antibody and one HCV RNA test, regardless of the screening strategy.

Since the share of symptomatic and linked-to-care cases varies by stage of liver disease, the input number of newly diagnosed cases were segmented by stage of liver disease, with the more advanced stages being diagnosed first. The modelled disease burden was used in this segmentation. It was also assumed that cases of spontaneously cleared HCV infection were diagnosed at the same rate as the asymptomatic chronically infected cases.

To calculate the size of the population eligible for screening, the module tracked the populations with a history of screening, diagnosis, or SVR. It was assumed that each person would receive at most one HCV antibody screen, and populations with a history of diagnosis or SVR would be excluded from future screening. Additionally, populations outside of the age range eligible for screening were excluded.

The number needed to screen (NNS) to find one HCV antibody-positive case was calculated as shown in Equations 1–2. Next, the number of annual HCV antibody screens performed was calculated as shown in Equation 3. Lastly, it was assumed that every newly diagnosed HCV antibody-positive case was followed up with an HCV RNA test. The economic impact of each screening strategy was calculated using the price of HCV antibody and HCVRNA screening tests, applied to the total number of anti-HCV and HCVRNA screens performed, respectively, in each scenario.

**Equation 1.** Number needed to screen to diagnose one HCV antibody-positive case, unadjusted, in year $t$

$\text{NNS}_{t}^{*}=\frac{1}{\frac{\text{Undiagnosed, asymptomatic or not linked-to-care HCV antibody-positive cases}_{t}}{\text{Population eligible for screening}_{t}}}$, where

$\text{Population eligible for screening}_{t}$was the population eligible for screening in year $t$. Eligibility of screening was determined by birth year (by scenario), history of having received a screen within the screening strategy (excluded), history of diagnosis (excluded), and history of SVR (excluded).

**Equation 2.** Number needed to screen to diagnose one HCV antibody-positive case, adjusted, in year $t$

$\text{NNS}_{t}=\frac{\text{NNS}_{t}^{*}}{k}$, where

$\text{NNS}_{t}^{*}$is the number needed to screen to diagnose one HCV antibody-positive case, unadjusted, in year $t$;

$k$is the ratio of prevalence in the population where the screening is conducted to that in the general population.

**Equation 3.** Number of HCV antibody screens performed, in year $t$

$\text{Newly diagnosed symptomatic HCV antibody-positive cases}_{t}+\text{NNS}_{t}\times\text{Newly diagnosed asymptomatic or not yet linked-to-care cases}_{t}$, where

$\text{NNS}_{t}$is the number needed to screen to diagnose one HCV antibody-positive case in year $t$.

**Appendix 2**

**Estimating the size of the PWID population, by birth cohort, and associated costs**

A standardized mortality ratio (SMR) of 10.0 (9.5–29.9) is applied in the model to the 15–44-year-old HCV-infected population, considering the estimated share of the HCV-infected population that is actively injecting. For Italy, the share of the HCV-infected population that is actively injecting was estimated as follows:

|  | **IDU Calculation** | **Total** | **Source** |
| --- | --- | --- | --- |
| $a$ | Population in 15–64 (in 2011) | 38,963,281 | 1 |
| $b$ | Prevalence of IDU in 15–64 (in 2011) | 0.38% | 1 |
| $\boldsymbol{c}$ | **Number of PWID in 15–64 (in 2011)** | **148,060** | $\boldsymbol{a\times b}$ |
| $d$ | HCV prevalence in PWID | 54% | 1 |
| $e$ | Viremic rate | 74.1% | 2 |
| $\boldsymbol{f}$ | **Total viremic PWID (15–64)** | **59,245** | $\boldsymbol{c\times d\times e}$ |
| $g_{1}$ | HCV-infected 15–64-year-olds | 466,600 | Model outcome |
| $\boldsymbol{h}_{\boldsymbol{1}}$ | **Share of HCV-infected population that are PWID (15–64)** | **12.7%** | $\frac{\boldsymbol{f}}{\boldsymbol{g}_{\boldsymbol{1}}}$ |
| **The share of PWID in the 55–64 cohort is assumed to be 50% of the 15–54 cohort (7.7% in 55–64; 15.4% in 15–54) | | | |

The average screening cost per person, considering the proportion of PWID in each cohort, was calculated as follows:

Equation 4. Proportion of PWID for scenario $S$, comprising birth years $a$ through $b$

$$\text{Proportion of PWID}_{S}=\frac{\sum_{i=a}^{b} \text{Cases in 2020}_{i}\times\text{\% PWID}_{i}}{\sum_{i=a}^{b} \text{Cases in 2020}_{i}}$$

For example, proportion of PWID for scenario $A$is$\frac{83,651\times0.0\%+75,410\times7.7\%+101,154\times15.4\%}{83,651+75,410+101,154}=8.2\%$.

Equation 5. Average screening cost per person for scenario$S$

$$\text{Average screening cost}_{S}=\text{Proportion of PWID}_{S}\times\text{Price PWID}+\left( 1-\text{Proportion ofPWID}_{S} \right)\times\text{Price non-PWID}$$

For example, average screening cost per person for scenario $A$is $8.2\%\times55+\left( 1-8.2\% \right)\times15=18$.

|  | Birth year, starting | Birth year, ending | Age range in 2020 | Cases in 2020 | % of cases in 2020 | % PWID | Cost PWID | Cost Non-PWID | Total Cost |
| --- | --- | --- | --- | --- | --- | --- | --- | --- | --- |
| 1 | 1935 | 1947 | 73–85 | 224,723 | 38% | 0.0% | 0 | 15 | 15 |
| 2 | 1948 | 1957 | 63–72 | 83,651 | 14% | 0.0% | 0 | 15 | 15 |
| 3 | 1958 | 1967 | 53–62 | 75,410 | 13% | 7.7% | 4 | 14 | 18 |
| 4 | 1968 | 1977 | 43–52 | 101,154 | 17% | 15.4% | 8 | 13 | 21 |
| 5 | 1978 | 1987 | 33–42 | 68,021 | 12% | 15.4% | 8 | 13 | 21 |
| 6 | 1988 | 2005 | 15–32 | 29,306 | 5% | 15.4% | 8 | 13 | 21 |
| 7 | 2006 | 2020 | 0–14 | 3,770 | 1% | 0.0% | 0 | 15 | 15 |
| A | 1948 | 1977 | 43–72 | 260,216 | 44% | 8.2% | 5 | 14 | 18 |
| B | 1958 | 1977 | 43–62 | 176,564 | 30% | 12.1% | 7 | 13 | 20 |
| C | 1948 | 1987 | 33–72 | 328,237 | 56% | 9.7% | 5 | 14 | 19 |
| D | 1935 | 2020 | 0–85 | 586,036 | 100% | 6.2% | 3 | 14 | 17 |

**Screening scenario inputs, 2018–2031**

The following table summarizes the estimated proportion of people who inject drugs and associated screening cost, per person, in each strategy.

| **GHSS elimination** | **<2020** | **2020–2022** | **2023–2025** | **% PWID†** | **Screening Cost (per person,** €**)** |  |
| --- | --- | --- | --- | --- | --- | --- |
| Screening 1948–1977 | Risk | 1948–1977 | 1948–1977 | 8.2% | 18 |  |
| Screening 1958–1977 | Risk | 1958–1977 | 1958–1977 | 12.1% | 20 |  |
| Graduated screening 1 | Risk | 1968–1987 | 1948–1967 | 9.7% | 19 |  |
| Graduated screening 2 | Risk | 1948–1967 | 1968–1987 | 9.7% | 19 |  |
| Universal screening | Risk | All ages | All ages | 6.2% | 17 |  |
| GHSS: Global Health Sector Strategy; PWID: Persons who inject drugs; Risk: risk-based screening assumes that those offered a test are approximately 5 times more likely to be infected than the general population  **†**See calculation in Appendix 2 [Appendix References 1,2] | | | | | | |

**References**

1. United Nations Office of Drugs and Crimes (UNODC). Statistics, People who inject drugs. 2017. Available from: https://data.unodc.org/

2. Andriulli A, Stroffolini T, Valvano MR, et al. Declining prevalence and increasing awareness of hepatitis C virus infection in Italy: a population‐based survey. *Eur J Intern Med*. 2018; **53**:79‐84.
